# A Micro-Force Sensor with Beam-Membrane Structure for Measurement of Friction Torque in Rotating MEMS Machines

**DOI:** 10.3390/mi8100304

**Published:** 2017-10-12

**Authors:** Huan Liu, Zhongliang Yu, Yan Liu, Xudong Fang

**Affiliations:** 1 Key Laboratory of Thin Film and Optical Manufacturing Technology, Ministry of Education, Xi’an Technological University, Xi’an 710032, China; 2 School of Mechanical Engineering and Automation, Northeastern University, Shenyang 110819, China; zhon.liangyu@gmail.com; 3 State Key Laboratory for Manufacturing Systems Engineering, Xi’an Jiaotong University, Xi’an 710049, China; ZTlover@stu.xjtu.edu.cn (Y.L.); dongfangshuo30@mail.xjtu.edu.cn (X.F.)

**Keywords:** beam-membrane structure, micro-force sensor, MEMS bearing, friction torque

## Abstract

In this paper, a beam-membrane (BM) sensor for measuring friction torque in micro-electro-mechanical system (MEMS) gas bearings is presented. The proposed sensor measures the force-arm-transformed force using a detecting probe and the piezoresistive effect. This solution incorporates a membrane into a conventional four-beam structure to meet the range requirements for the measurement of both the maximum static friction torque and the kinetic friction torque in rotating MEMS machines, as well as eliminate the problem of low sensitivity with neat membrane structure. A glass wafer is bonded onto the bottom of the sensor chip with a certain gap to protect the sensor when overloaded. The comparisons between the performances of beam-based sensor, membrane-based sensor and BM sensor are conducted by finite element method (FEM), and the final sensor dimensions are also determined. Calibration of the fabricated and packaged device is experimentally performed. The practical verification is also reported in the paper for estimating the friction torque in micro gas bearings by assembling the proposed sensor into a rotary table-based measurement system. The results demonstrate that the proposed force sensor has a potential application in measuring micro friction or force in MEMS machines.

## 1. Introduction

The micro gas bearing is frequently used as a very important component for rotating micro-electro-mechanical system (MEMS) devices that can carry load in micro-rotating machinery [1]. Compared to macro gas bearings, MEMS-based bearings work at a higher speed and require better repeatability and stability. Current studies mainly investigate the design and fabrication [2,3], and slip effect [4,5] of gas bearings, but little attention is given to the characterization of the friction torque. Investigating the friction torque of gas bearings can help control lubrication flow and propose a more effective design scheme. However, accurate calculation of friction torque is still a challenge [6,7,8], and performing experimental analysis inevitably brings forth a favorable solution to the investigation of micro-friction torque in MEMS gas bearings.

Because of the very small size and target parameters in the gas bearing, conventional methods cannot provide enough accuracy, both in measurement and installation precision [9,10]. Therefore, the authors set up a rotary table-based measurement system to accomplish the task. The friction torque can be transformed into the more easily detected force parameter by a force arm, and the rotary table with necessary gas passage are all available in the laboratory. Then the force sensor becomes the key device for the system. As mentioned above, the sensor should possess a large measuring range to enable its usage in the measurement of different friction status (dry or lubricated), a good sensitivity to accurately detect the target force, and an overload protection to avoid unexpected breakage during sensor installation or incorrect experiments.

The literature shows that several MEMS force-sensing devices have been developed. The beam structure and membrane structure are typically used in the sensing configuration [11,12]. The membrane sensor features a relatively large measurement range, but its high supporting stiffness inevitably decreases the measurement sensitivity, which places obstacles in the sensor applications; though reducing the thickness of membrane can tackle this problem, thin membrane may increase the difficulty in sensor fabrication and preserving dimensions accuracy. The beam structure is simply constructed with high sensitivity, but the measurement range is limited by the stiffness of the beams and low linearity may appear when the force-induced deflection is large [13]. A. Jordan and S. Büttgenbach [14] utilize SU-8 resistance to develop a membrane force sensor with low bending stiffness, but it is difficult to fabricate and the low admissible stress of resistance may also limit the range for force measurement. G. Palli and S. Pirozzi [15] developed an optical force sensor and it is very effective in measuring the tendon tension, but the inherent complexity makes it hard assemble with the torque measurement system. Yoshikawa et al. [16] reported a nanomechanical membrane-type surface stress sensor which provides high accuracy, but the planar scheme is unsuitable for contact force measurement.

This paper aims to provide an available solution to the force sensor with relatively large measurement range (0–40 mN) and favorable sensitivity (0.1 mV/mN), which can be used in the friction torque measurement system. The requirements for the sensors are a measurement range of 40 mN and a sensitivity of about 0.1 mV/mN, considering the to-be-measured force, unexpected collision in the operation and conditions in the laboratory. Focused on these target performances, a piezoresistive force sensor with beam-membrane structure is proposed. Finite element models for the conventional beam structure and membrane structure are established to gain insight into the influence of configuration on the sensor performances and an improved design attempt is based on the analysis results. The sensing structural design, piezoresistors arrangement, probe choosing and overload protection are all conducted in the design section. Then, the fabricated sensor prototype are packaged and calibrated, and a practical friction torque measurement is performed to verify the feasibility of utilizing the developed sensor in the measurement system.

## 2. Design

**Figure 1:**
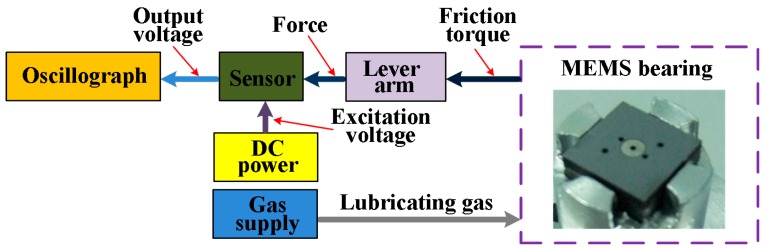
The schematic diagram of the measurement system for friction torque measurement.

The schematic diagram of a measurement system for friction torque in the MEMS gas bearings is illustrated in Figure 1. In the system, the to-be-measured torque is transformed into a force by the lever arm fixed onto the shaft of bearings. The force can be sensed by the relevant piezoresistive sensor, the output voltage of which is acquired by the oscillograph building up the variation curve of friction torque during the rotation of the bearings. The excitation voltage for the Wheatstone bridge is provided by a direct current (DC) power supply and the gas supply is used to provide the lubricating gas for the bearings. The friction torque in the MEMS gas bearings is in the 0–100 µN·m scale [17], and the fragile sensor structure may be broken during the system assembling. Therefore, the design of the sensor for the system should take into account the following specifications:

- The sensor should have a relatively high sensitivity to sense the weak force signal. Though a short lever arm can enlarge the force applied to the sensor, the arm installation and alignment between the lever arm and the sensor may turn into a hard connection.
- Excellent linearity is important. The strain in the sensing region should be less than 1/6–1/5 of the material’s ultimate strain, so as to ensure the measurement linearity, namely less than 500 μm for the silicon [18].
- Overload protection is required. Since the silicon-based sensors are fragile, unpredictable impulsive force in sensor packing, system assembling or practical measuring can break the structure, putting the sensor out of work.
- A proper sensor form is needed. The sensor will be connected to the lever arm to measure the target force, and a well-designed form can make the connection more easy and precise.

The first two points are mainly determined by the sensing geometry and structural dimensions, the latter two are related to the design of sensor components. Looking at the existed components available in the literature, the probe is often used to transmit the force from measurement target to the sensing mechanism and overload protection is implemented by bonding the glass or silicon wafer to limit the displacement range of central mesa [11]. Therefore, the probe and boned wafer are also selected as the elements in the proposed sensor. In the following part of this section, the investigation of two common structures, namely beam structure and membrane structure, will be performed, the design of new sensing structure for the force sensor based on the analysis results will be presented and the arrangement of probe and piezoresistors are also conducted.

### 2.1. Design of Sensor Geometry

**Figure 2:**
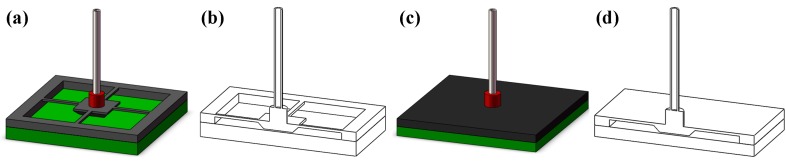
Beam structure (**a**,**b**) and membrane structure (**c**,**d**) for the force sensor.

In the design of sensing structure for force sensors, the beam structure and membrane structure are the most commonly used geometry in the published literature. Figure 2 is a schematic view of the two structures. The two structures are chosen to analyze the influence on the sensor performance from the geometry. The beam structures, shown in Figure 2a,b consist of four identical beams supporting the central mesa, on which a probe is adhered with a basement. The membrane structure has a similar configuration, but the supporting structure changes into a membrane (shown in Figure 2c,d).

**Figure 3:**
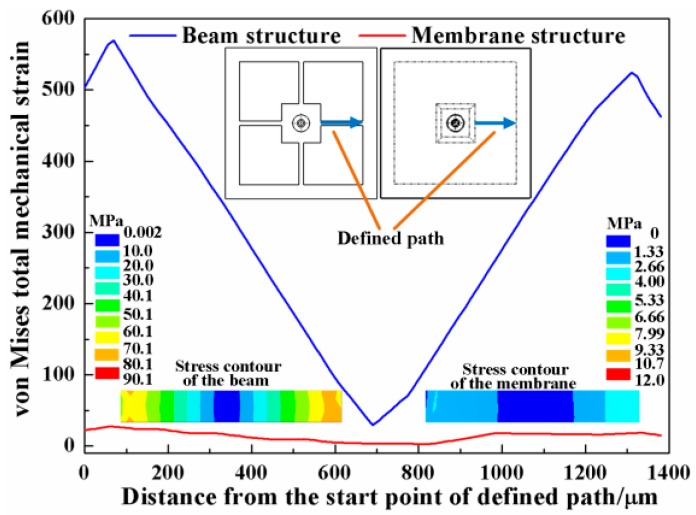
The strain distribution and stress contour of beam and membrane structures.

To analyze the performance of the two common structures under the applied force of 40 mN, finite element (FE) models are established. The material parameters of the silicon are set as Young’s modulus E = 166 GPa, density ρ = 2331 kg/m^3^ and Poisson’s ratio ν = 0.278. The overall dimension of the chip is 7000 μm × 7000 μm, with a 2300 μm width mesa. In the beam structure, the length, width and thickness of the four beams are 1380 μm, 200 μm and 50 μm, respectively; in the membrane, the membrane covers all of the space between the outer frame and central mesa with thickness of 50 μm. The force is applied symmetrically on the tip of the probe at two points and the outer frame is rigidly fixed. Figure 3 is the strain distribution and stress contour along the defined path in the beam and membrane structures. Under the same force load, the beam structure acquires larger strain, especially at the two ends. The strain comes over 500 με, which may greatly affect the linearity of the sensor output. Meanwhile, the membrane structure only has a strain of about 20 με, which is too small for the high-sensitivity-needed force sensor.

**Figure 4:**
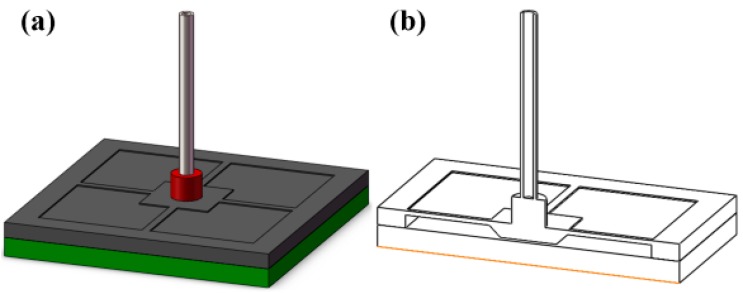
The designed beam-membrane structure. (**a**) 3D view; (**b**) cross-sectional view.

**Figure 5:**
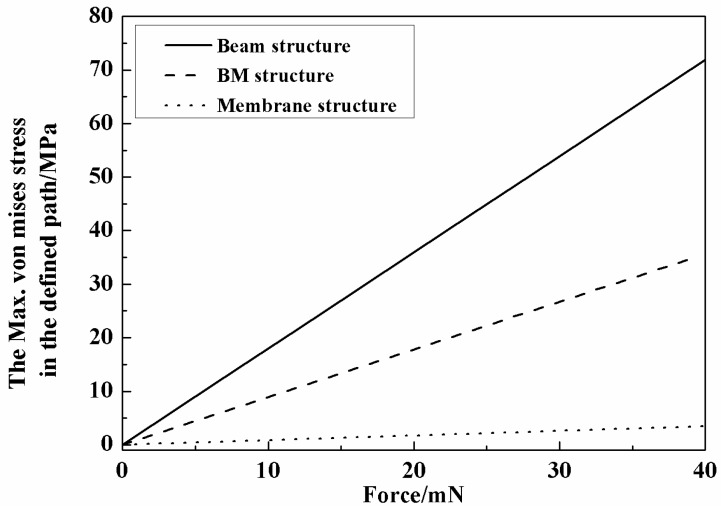
Sensitivity comparison of three structures.

**Figure 6:**
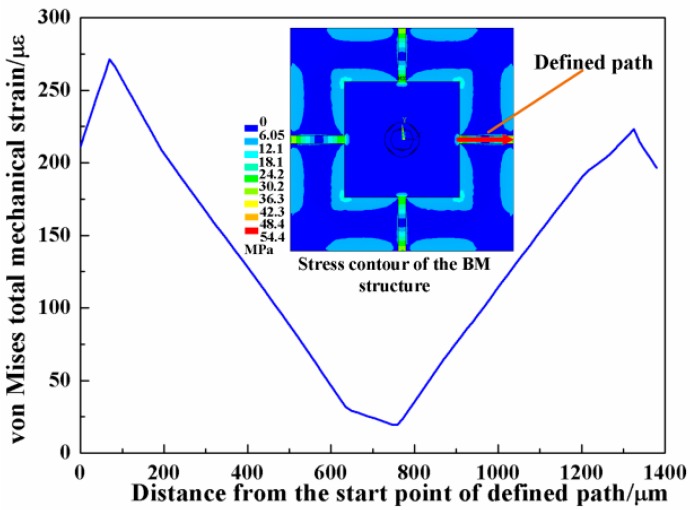
The strain distribution and stress contour of beam-membrane structure.

The stiffness of the beam structure is not enough for the target force and the membrane is too rigid to sense the force. Thus, a combination of the two structures, namely the beam-membrane (BM) structure shown in Figure 4, can be an effective solution for the high sensitivity force sensor. In the newly-designed geometry, four beams are settled atop the membrane, connecting with the central mesa along the central lines of the mesa. The BM structure utilizes the membrane to enhance the supporting stiffness, while the stress accompanying with the force load will concentrate on the beams, which ensures that the sensor still has a favorable sensitivity. In the BM structure, the thickness of the membrane is 30 μm, and the other dimensions are the same to the abovementioned membrane structure; for the size of beams, thickness is also the only changed parameter that turns into 20 μm. To compare the three structures, the FE model of BM structure is also established and the simulated relationships between the maximum von Mises stress in the beam/membrane and applied force are shown in Figure 5. It can be found that all the structures have a good linearity relationship between the force and the accompanying stress, and the BM structure has a more favorable stress that maintains measurement sensitivity and keeps the total strain below 500 με to avoid possible nonlinear deformation in the sensing region. The strain distribution of the BM structure is shown in Figure 6, and the maximum total strain of the beam in is about 275 με, conforming to the linearity requirement.

### 2.2. Design of Sensor Components

As the signal transforming component in the sensor, piezoresistors play an important role in the force measurement. For a piezoresistor oriented in [110] direction, the variation of the resistance can be expressed as Δ*R* [19].

(1) $$\frac{\Delta R}{R}=\frac{1}{2}\pi_{44}\left(\sigma_{l}-\sigma_{t}\right)$$

**Figure 7:**
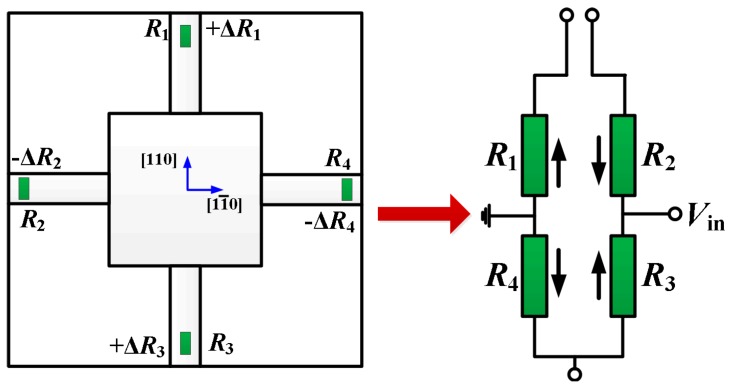
The piezoresistors arrangement in the beam-membrane (BM) structure sensor.

In Equation (1), *R*, Δ*R* are the original and variation resistance of the piezoresistor, *σ*_1_, *σ*_t_ are the stresses in the longitudinal and transverse directions of the piezoresistor, *π*_44_ is the shearing piezoresistance coefficient. The piezoresistors arrangement is shown in Figure 7, for *R*_1_ and *R*_3_ the beam normal stress are the longitudinal stress and they will obtain a positive change; meanwhile, the normal stress turns into transverse stress for *R*_2_ and *R*_4_, and their variation will be negative. Therefore, a full Wheatstone bridge can be constructed by the four piezoresistors, as Figure 7 shows.

Another component is the sensor probe. The probe should be inflexible and lightweight to eliminate the influence on the sensor performances from the incorporation of probe. Therefore, a tubular shape, stainless-steel-made probe is chosen. Moreover, a sleeve is used to enhance the installation accuracy, which is marked red in Figure 4. According to the FE analysis, the maximum displacement of the central mesa is 3 μm when the 40 mN force is applied. Therefore, to protect the sensing structure from broken, the Pyrex glass wafer is attached on the bottom of the chip with a gap of 3 μm from the bottom of the central mesa.

## 3. Sensor Realization

### 3.1. Fabrication

**Figure 8:**
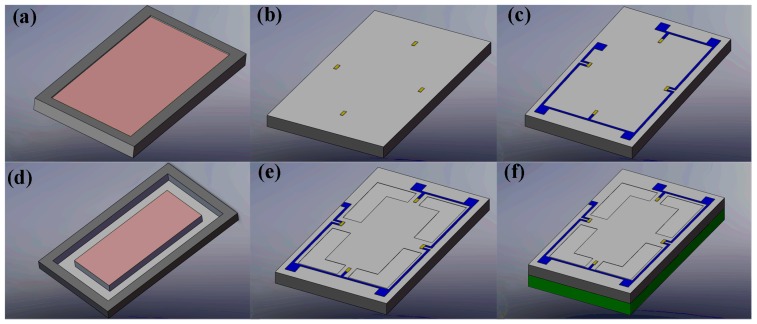
Main fabrication process of the sensor chip. (**a**) Etching at the back side to form the gap between the bottom of the central mesa and bonded glass; (**b**) Boron ion diffusion and driven-in process for piezoresistors on the front side; (**c**) Al sputtering to form the interconnections of piezoresistors and the electrodes; (**d**) The central mesa was shaped by wet etching from the back side; (**e**) The beam-membrane structure was formed by inductive coupled plasma (ICP) etching on the front side; (**f**) Pyrex glass wafer was bonded onto the bottom of silicon wafer by anodic bonding.

The main fabrication process of the sensor chip is shown in Figure 8. A double-side polished, N-type (100)-oriented silicon wafer was used as the start material and a total of eight masks were needed. The detailed fabrication process is as follows:

(1) The fabrication began with the inductively coupled plasma (ICP) etching at the back side to form the working gap between the bottom of the central mesa and bonded glass (Figure 8a);
(2) Then the boron ion diffusion and driven-in process were conducted after the piezoresistors were patterned on the front side (Figure 8b);
(3) The interconnections of piezoresistors were realized by Al sputtering and the electrodes were simultaneously formed (Figure 8c);
(4) The deposited Si_3_N_4_ and SiO_2_ were utilized as the protection layer for the following KOH etchant based anisotropic wet etching, and the central mesa was shaped by wet etching from the back side (Figure 8d);
(5) The beam-membrane structure was formed by ICP etching on the front side (Figure 8e);
(6) A Pyrex glass wafer was bonded onto the bottom of silicon wafer by anodic bonding (Figure 8f). A Cr/Au bi-layer was deposited on the glass wafer to avoid electrostatic adhesion between the mesa and glass during the bonding or overloaded process.

**Figure 9:**
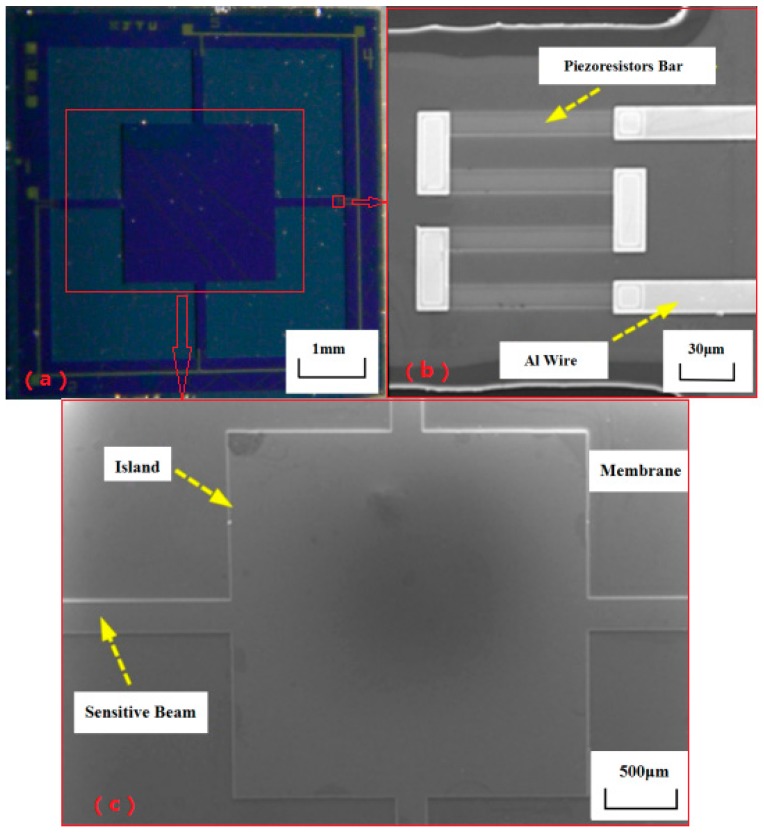
The fabricated sensor chip. (**a**) Photo of whole chip (**b**) SEM of piezoresistors and Al wire (**c**) SEM of the beam, membrane and island (the base of probe).

The finished sensor chip is shown in Figure 9.

### 3.2. Sensor Packing

**Figure 10:**
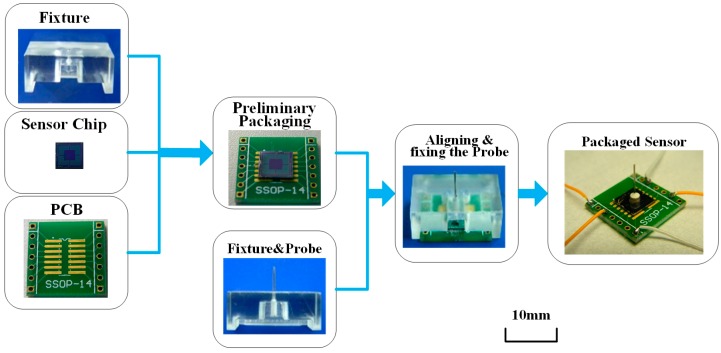
Packaging of the force sensor.

To guarantee the precision of the packaging, a tailor-made organic glass fixture is designed. The fixture has two grooves for the positioning of the sensor and printed circuit board (PCB), and a hole for the probe. The packaging process is illustrated in Figure 10. Firstly, the sensor chip and PCB were aligned with the grooves in the glass fixture and bonded by adhesive; after that, the probe was put into the hole and adhered onto the central mesa of the sensor chip; finally, the electrical connection between the sensor and instruments was conducted with golden leads by the wire bonder after the necessary thermal solidification of adhesive.

## 4. Sensor Testing 

### 4.1. Experimental Setup

**Figure 11:**
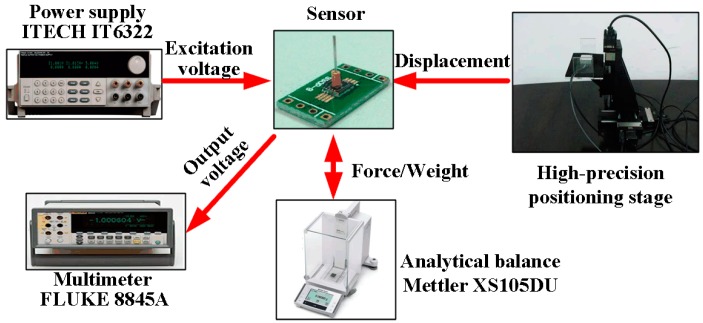
The setup for sensor calibration.

The calibration system, shown in Figure 11, uses an analytical balance and high-precision positioning stage to generate and display the input force for the sensor; the force can be obtained by multiplying the measured weight and gravity acceleration (10 m/s^2^). The positioning stage consists of a tri-dimensional positioning stage and a piezoelectric ceramics with the resolution up to nm scale. The tri-dimensional positioning stage adjusts the sensor location, making the tip of the sensor probe very close to the sensing plate of balance; then, the piezoelectric ceramics moves the sensor with nm displacement, pressuring the sensor probe to the balance. The excitation voltage for the Wheatstone bridge in the sensor is provided by the power source (ITECH IT6322, ITECH Electronic Co., Ltd., Nanjing, China), and the output voltage can be measured by a digital multimeter (FLUKE 8845A, Fluke Inc., Everett, WA, USA).

### 4.2. Results and Discussion

**Figure 12:**
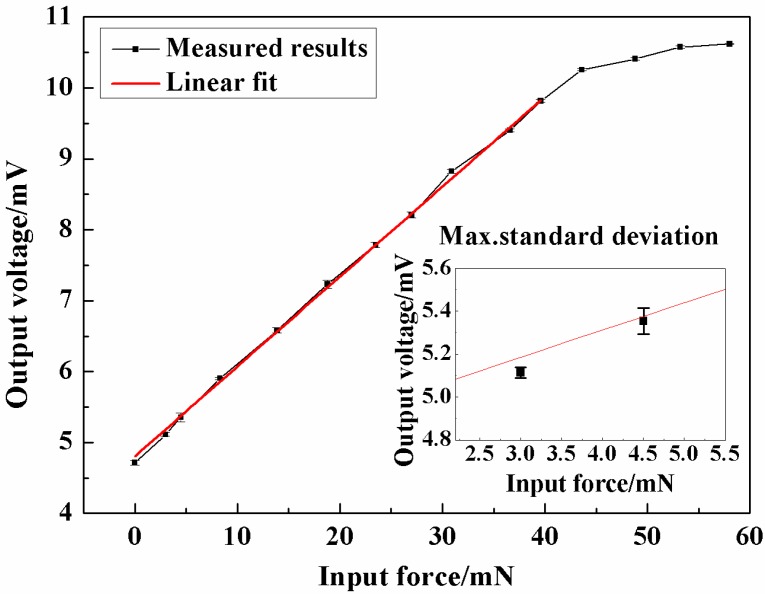
The results of sensor calibration and overload protection.

The static characteristics of the force sensor were tested by the abovementioned calibration system. The excitation voltage for the Wheatstone bridge was 5 V DC and the applied force rose from 0 to about 50 mN and decreased to 0 in one test. The experimental results were shown in Figure 12. When the applied force is larger than 40 mN, the increase tendency of output voltage became tardy and the protection glass at the bottom of the sensor chip began to play its role. In the designed linear range of 0–40 mN, the results were fitted with the least square method and the relationship between the applied force *F* (mN) and output voltage *V* (mV) can be expressed as Equation (2) as follows:

(2) V=0.127F+4.805

Obviously, the measured sensitivity was 0.127 mV/mN, and the maximum non-linearity was 1.97 %FS and the total measurement accuracy is 2.13 %FS. The main goal of a measurement range of 40 mN and a sensitivity of about 0.1 mV/mN are achieved. The other tested parameters of the sensor are shown in Table 1.

## 5. Sensor Application 

### 5.1. Experimental Setup

**Figure 13:**
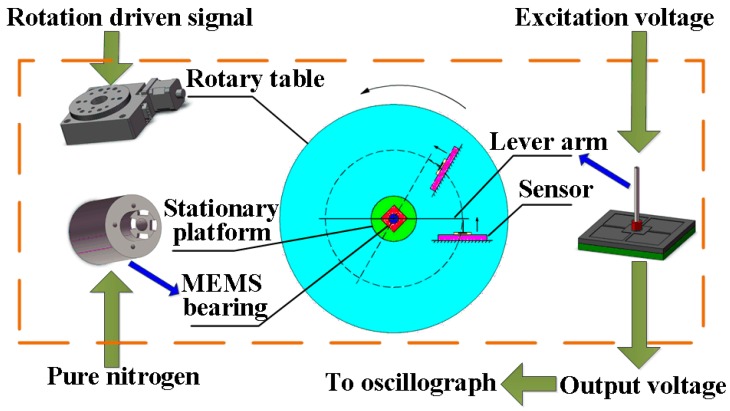
The system for friction torque measurement in micro-electro-mechanical system (MEMS) bearing.

The actual measurement system is sketched in Figure 13. The ultra-precision computer-controlled rotary table generates the original rotation by its step motor, which is transmitted to the shaft of the gas bearings by the force sensor and a silicon-based lever arm. The lever arm is fabricated by laser scribing apparatus, and the final size is 50 mm long, 1 mm wide and 400 µm thick. The sensor probe is horizontal and vertically connected to the lever arm which is fixed onto the shaft of MEMS bearing.

**Figure 14:**
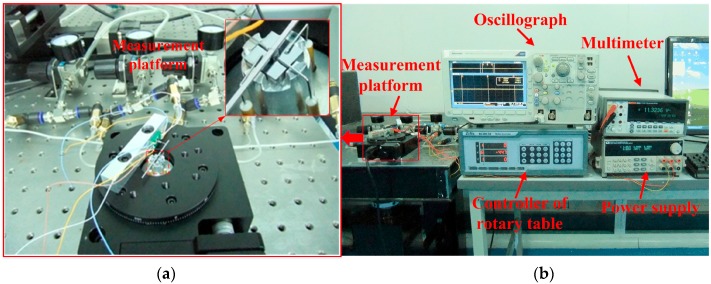
Photo of the measurement platform (**a**) and the assembled system (**b**).

The MEMS bearing is installed in the central hole of the rotary table by a stationary platform with necessary gas passage. The force sensor is adhered on an off-center settled fixture with an adjustable distance from the table center. The whole platform is fixed onto a horizontal stand. The assembled system is shown in Figure 14. High purity nitrogen is led to the gas bearings for suspension and lubrication. The gas pressure for upper gas bearing is 4.5PSI, while 4.86PSI for the lower gas bearing to balance the weight of the lever arm, shaft and rotor. The length of force arm for the lever and sensor is set as 5 mm which can be changed by moving the connection point between the sensor probe and lever arm.

Running scheme of the rotary table is programmed to rotate from stillness to uniform rotation at the speed of 1°/s. The output voltage of the force sensor was recorded by an oscillograph and shown in Figure 14b. The maximum static friction torque occurs when the maximum voltage is output during the starting process.

### 5.2. Results and Discussion

**Figure 15:**
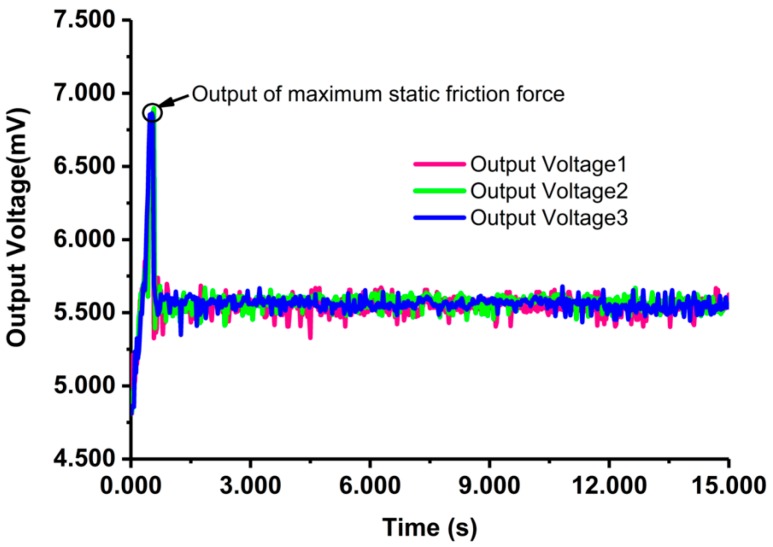
The output voltage of the sensor under start-up and steady stage.

As shown in Figure 15, the maximum voltage is obtained at the end of the pulse-on process. Correspondingly, the maximum starting friction torque is achieved with the maximum starting friction force at this time. Three representative curves are collected with the maximum voltage of 6.856 mV, 6.845 mV and 6.862 mV, respectively. The average value of the maximum voltages is 6.854 mV and the matching force is 16.314 mN, which is calculated according to the calibration results in Figure 14. Hence, the maximum starting friction torque is 80.669 μN·m, considering the length of the force arm is 5 mm.

Passing the pulse-on process, the bearings began to set up uniform rotation and the output of the force sensor maintained in a stable-state range. The average voltage is 5.559 mV for the three representative curves in the steady stage. The matching friction force is 5.937 mN and the corresponding torque is 29.685 μN·m.

As observed from the figure, the three curves show high repeatability, only with small fluctuations at steady stage. Possible reasons for the fluctuation can be unstable gas flowing in the bearing or gas turbulence in the engine [20].

With the above results, it is demonstrated that the measurement range and sensitivity of the sensor we designed and fabricated can meet the requirements for measuring large starting friction torque and kinetic friction torque in rotating MEMS machines. This sensor, which incorporates a membrane into conventional four-beam structure, has the capability to eliminate the problem of small measurement range with neat beam structure and low sensitivity with neat membrane structure.

## 6. Conclusions

A force sensor with beam-membrane structure has been designed, fabricated and tested for use in a friction torque measurement system. The proposed sensing structure combines the conventional beam structure and membrane structures, which gives the sensor a relatively high sensitivity and high reliability. A hollow stainless steel probe is bonded to the fabricated sensor chip with the help of a tailor-made glass fixture. According to the results of sensor calibration, the sensitivity, maximum non-linearity error and measurement accuracy of the developed sensor are 0.127 mV/mN, 1.97 %FS and 2.13 %FS, respectively. The bonded glass wafer at the bottom of the sensor chip can act as overload protection when the applied force is beyond the designed range. With the aim of evaluating the usage of the force sensor in the system for friction torque measurement, practical experiments for estimation of friction state in the gas bearings are conducted. The results show promising variation tendency of friction in the gas bearing, demonstrating the feasibility of embedding a MEMS force sensor into the measurement system to investigate the friction torque down to the scale of 100 μN·m. Therefore, the sensor has great potential application in in-situ measuring micro friction force and torque under pulse-on stage and stable stage in rotating MEMS machines.

## Figures and Tables

**Table 1:** Performance of the beam-membrane (BM) micro-force sensor.

Parameter	Value
Zero point offset (mV)	4.805
Full scale span (mV)	9.885
Sensitivity (mV/mN)	0.127
Nonlinearity (%FS)	1.97
Temperature Drift Factor (/°C)	−0.00628
Time Drift (%FS/h)	0.30473
Hysteresis (%FS)	0.548
Repeatability (%FS)	0.761
Accuracy (%FS)	2.13
